# Assessing *Acanthamoeba* cytotoxicity: comparison of common cell viability assays

**DOI:** 10.3389/fmicb.2023.1175469

**Published:** 2023-04-25

**Authors:** Alvie Loufouma Mbouaka, Iwona Lesiak-Markowicz, Irene Heredero-Bermejo, Rounik Mazumdar, Julia Walochnik, Tania Martín-Pérez

**Affiliations:** ^1^Center for Pathophysiology, Infectiology and Immunology, Institute of Specific Prophylaxis and Tropical Medicine, Medical University of Vienna, Vienna, Austria; ^2^Department of Biomedicine and Biotechnology, Faculty of Pharmacy, University of Alcalá, Madrid, Spain; ^3^Max Perutz Labs Vienna, Department of Medical Biochemistry, Medical University of Vienna, Vienna, Austria

**Keywords:** *Acanthamoeba*, human corneal epithelial cells, pathogenesis, amoeba-host cell interaction, viability, cytotoxicity

## Abstract

**Background:**

*In vitro* models for studying interactions between *Acanthamoeba* and host cells are crucial for understanding the pathomechanism of *Acanthamoeba* and assessing differences between strains and cell types. The virulence of *Acanthamoeba* strains is usually assessed and monitored by using cell cytotoxicity assays. The aim of the present study was to evaluate and compare the most widely used cytotoxicity assays for their suitability to assess *Acanthamoeba* cytopathogenicity.

**Methods:**

The viability of human corneal epithelial cells (HCECs) after co-culture with *Acanthamoeba* was evaluated in phase contrast microscopy.

**Results:**

It was shown that *Acanthamoeba* is unable to considerably reduce the tetrazolium salt and the NanoLuc^®^ Luciferase prosubstrate to formazan and the luciferase substrate, respectively. This incapacity helped to generate a cell density-dependent signal allowing to accurately quantify *Acanthamoeba* cytotoxicity. The lactate dehydrogenase (LDH) assay led to an underestimation of the cytotoxic effect of *Acanthamoeba* on HCECs since their co-incubation negatively affected the lactate dehydrogenase activity.

**Discussion:**

Our findings demonstrate that cell-based assays using the aqueous soluble tetrazolium-formazan, and the NanoLuc^®^ Luciferase prosubstrate products, in contrast to LDH, are excellent markers to monitor the interaction of *Acanthamoeba* with human cell lines and to determine and quantify effectively the cytotoxic effect induced by the amoebae. Furthermore, our data indicate that protease activity may have an impact on the outcome and thus the reliability of these tests.

## 1. Introduction

*Acanthamoeba* spp. are ubiquitous free-living amoebae occurring worldwide in water environments and soil, but they can also be isolated from dust and the air. They are facultative pathogens and can cause different diseases, importantly, the so-called *Acanthamoeba* keratitis (AK), a severe infection of the cornea, most commonly observed in contact lens wearers. The incidence of AK has increased within the past decades, which may be attributed to the increasing number of contact lens users, but also to advances in diagnostics (Chawla et al., 2014; Randag et al., 2019; List et al., 2021). *Acanthamoeba* spp. may also cause granulomatous amoebic encephalitis, a fatal disease of the central nervous system, and other disseminating infections, mainly in immunocompromised individuals (Morrison et al., 2016). Treating these infections is challenging, as no specific drugs are currently available (Siddiqui et al., 2014; Lorenzo-Morales et al., 2015; Loufouma Mbouaka et al., 2021).

The pathomechanism of these amoebae still remains incompletely understood, despite significant progress made in recent years (Clarke and Niederkorn, 2006; Khan, 2006; Walochnik and Duchêne, 2016). Adhesion to the host cells is mediated by a mannose-binding protein (MBP) and other adhesion proteins, triggering the release of proteases, mainly of the serine and metalloprotease type. The immune reaction of the host is characterized by neutrophil migration and macrophage activation resulting in the release of proinflammatory cytokines such as tumor necrosis factor alpha (TNF-α), interleukin-1β (IL-1β), and interleukin-6 (IL-6). In AK, secretory IgA antibodies in the tears can prevent binding of the *Acanthamoeba* trophozoites (Mattana et al., 2002; Garate et al., 2004; Walochnik and Duchêne, 2016). However, only a comparably small percentage of environmental *Acanthamoeba* isolates are able to lyse human cells and cytopathogenicity is known to decline during long-term axenic culture – but can also be enhanced by mouse passage or serial passage over human cell lines (Mazur and Hadaś, 1994; Koehsler et al., 2009). Cell viability and cytotoxicity assays are useful tools to determine the cytotoxic effects of *Acanthamoeba* on human cells because they measure *in vitro* modifications at the cellular and metabolic levels by detecting structural changes such as loss of membrane integrity or physiological and biochemical responses associated with non-viable and viable cells, respectively (Riss et al., 2004). They are typically used in drug discovery screening to assess the effect of a compound on cell proliferation (Riss et al., 2004). However, depending on the research aims and owing to the limitations of these assays, their use may be challenging. Recently, it was demonstrated that some of these assays, when used to study and evaluate host–pathogen interactions, may interfere with the pathogen and lead to an inaccurate estimation of pathogen cytotoxicity and their effects on host cells during and after co-culture (Van den Bossche et al., 2020). Thus, it is crucial to identify or develop and validate reliable methods and models to study such interactions and to avoid any interference with the culture medium or the pathogen in co-culture.

The present study aimed to evaluate and compare the most widely used cell viability assays for their usefulness to assess the cytotoxicity of *Acanthamoeba* spp. on human corneal epithelial cells (HCECs) during co-culture. Cell morphology, viability and integrity were evaluated by phase contrast microscopy.

## 2. Materials and methods

### 2.1. *Acanthamoeba* strains

The non-pathogenic environmental isolate strain *Acanthamoeba castellanii* Neff (ATCC 50373) and two pathogenic isolates from patients with keratitis, strains 1BU and strain SIN20, isolated in 1998 and 2020, respectively, all belonging to the T4 genotype group, were used in this study. The strains were maintained on non-nutrient agar plates coated with *Escherichia coli*. Prior to the experiments, all strains were sub-cultured and grown axenically at 34°C in peptone, yeast extract, and glucose (PYG) medium containing 10 g proteose peptone, 10 g yeast extract, 5 g NaCl, 5 g glucose, 0.7 g Na_2_HPO_4_, and 0.7 g KH_2_PO_4_ per liter.

### 2.2. Human corneal epithelial cells (HCECs)

Cells and media components were purchased from Innoprot (Derio, Bizkaia, Spain). Immortalized human corneal epithelial cells (HCECs; P10871-IM) were sub-cultured in corneal epithelial cell medium (CEpiCM, P60189) containing the 5% fetal bovine serum (FBS), 1% epithelial cell growth supplement (ECGS) and 1% penicillin/streptomycin at 37°C and 5% CO_2_. T75 flasks and 96-well plates were coated with a thin layer of type I collagen (P8188) to enhance cell attachment and proliferation. Prior to the assays, different concentrations of HCECs were assessed to determine the optimal concentration for the subsequent experiments.

### 2.3. *Acanthamoeba*–HCEC co-culture

At least three independent experiments were performed in triplicates. Before the assays, 1 × 10^4^ HCECs per well were seeded in a 96-well plate and incubated overnight. Then, the medium was replaced with a serum-free medium, and cells were maintained at 34°C and 5% CO_2_ during the co-culture to mimic the conditions of the human eye. Amoebae were added into the wells at different ratios or multiplicity of infection (MOI 1, MOI 2, and MOI 3; see Table 1) and incubated for 2, 4, 6, and 8 h. Wells containing only the HCECs were considered as positive controls, with a percentage of viability close to 100% for the following assays.

**TABLE 1:** Multiplicity of infection (MOI) used for cytotoxicity assays.

MOI	Number of amoeba*	Number of HCEC*
1	1.0 × 10^4^	1.0 × 10^4^
2	2.0 × 10^4^	1.0 × 10^4^
3	3.0 × 10^4^	1.0 × 10^4^

*The total volume of the experimental serum-free medium per well containing either amoeba alone, in co-culture with HCECs or HCECs alone was 100 μl.

### 2.4. Cell viability and cytotoxicity assays

#### 2.4.1. Lactate dehydrogenase assay

Lactate dehydrogenase (LDH) assay is a colorimetric method used to assess cytotoxicity and quantify cell viability. The damage to the plasma membrane allows the release of LDH from the intracellular environment into the cell culture medium and can be quantified using a coupled enzymatic reaction. The LDH activity was determined using the CyQUANT™ LDH Cytotoxicity Assay Kit (Thermo Fisher Scientific, Eugene, OR, USA), and strictly following the manufacturers’ instructions (Thermo Fisher, 2019). Simultaneously, the LDH positive control was established by adding the same volume of lysis buffer to samples containing only HCECs or *Acanthamoeba* alone. The plate was incubated at room temperature and protected from light for 30 min. Then, the absorbance was measured at 490 nm using a microplate absorbance spectrophotometer (Anthos Labtec Instruments HT2, Salzburg, Austria). The percentage of cytotoxicity was determined following the formula provided with the LDH assay kit and then converted to relative cell viability.

#### 2.4.2. MTS assay

The MTS assay is a colorimetric method used to assess cell viability. Viable cells reduce the yellow tetrazolium compound [3-(4,5-dimethylthiazol-2-yl)-5-(3-carboxymethoxyphenyl)-2-(4-sulfophenyl)-2H-tetrazolium, inner salt; MTS] to soluble formazan, which is purple. CellTiter 96^®^ AQ_*ueous*_ One Solution Reagent (Promega, Madison, WI, USA) was added to each sample (20 μl per well) in a 96-well plate, and the plate was incubated at 34°C and 5% CO_2_. After 1 h incubation, absorbance at 490 nm was determined using a spectrophotometer (Tecan, Spark 10M, Switzerland).

#### 2.4.3. RealTime-Glo™ MT Cell Viability assay

The RealTime-Glo™ MT Cell Viability assay (Promega, Madison, WI, USA) is a bioluminescence assay used to assess cell viability in real time. Metabolically active cells reduce the NanoLuc^®^ luciferase prosubstrate to the luciferase substrate, thus producing a luminescence signal, which correlates with the number of viable cells. During this assay, an opaque-walled tissue culture plate was used. The 2X RealTime-Glo™ reagent was prepared with serum-free CEpiCM, and 50 μl was added into each well containing HCECs, followed by inoculation with an equal volume of serum-free medium containing amoebae in suspension at different MOIs. The plate was placed in a cell culture incubator at 34°C and 5% CO_2_, and cell viability was measured every 2 h. The viability was assessed in real time over 8 h, using a plate-reading luminometer (Tecan, Spark 10M, Switzerland).

### 2.5. Microscopy

All cells were analyzed by phase contrast microscopy and trypan blue staining. Trypan blue facilitates the determination of the cell number and percentage of viability within a cell population (Riss et al., 2004; Strober, 2015). A μ-slide 8-well chamber (Ibidi, Martinsried, Germany) coated with collagen I was used for microscopic observation and analysis. HCECs (5 × 10^3^ per well) were seeded in the chamber and incubated overnight. After approximately 16 h, the medium was replaced with a serum-free medium, and amoebae were added at different MOI as previously described; the plates were incubated for different time periods (2, 4, 6, 8, and 24 h). For phase contrast microscopy and microphotography, a Nikon Eclipse TE200 microscope with NIS-Elements version 4.00.07 software (Optoteam, Vienna, Austria) was used.

### 2.6. Statistical analysis

Data was analyzed through two-way ANOVA with Dunnett’s multiple comparisons test using GraphPad Prism version 7.0 for Windows (GraphPad Software Inc, San Diego, CA, USA).

## 3. Results

### 3.1. Growth of *Acanthamoeba* under various conditions

**FIGURE 1:**
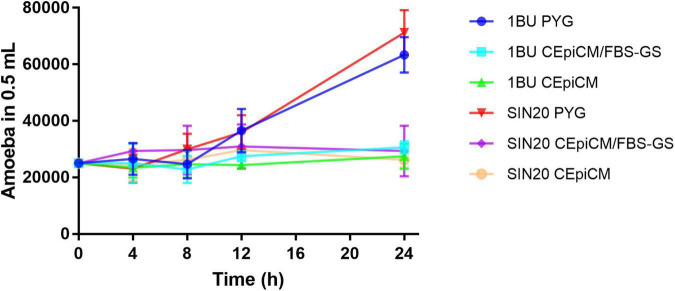
Growth curves of *Acanthamoeba* strains 1BU and SIN20 under various conditions over time at 34°C with 5% CO_2_ atmosphere. The numbers of amoebae were determined by cell counting with a hemocytometer.

To ensure cell integrity, *Acanthamoeba* SIN20 and 1BU were monitored under various conditions, using CEpiCM with serum, serum-free CEpiCM, and PYG medium. Under these conditions, in serum-containing and serum-free CEpiCM, the number of amoebae on both strains remained constant over time for up to 24 h (Figure 1). Under all conditions, no dead cells were observed during the experiments. In PYG medium, the number of amoebae slightly increased after 8 h of incubation, followed by a significant increase after 12 h (Figure 1). Therefore, the time point of 8 h was selected as the maximal duration for all cytotoxicity assays, also to maintain the defined MOI ratio.

### 3.2. *Acanthamoeba* cell density affected lactate dehydrogenase production

**FIGURE 2:**
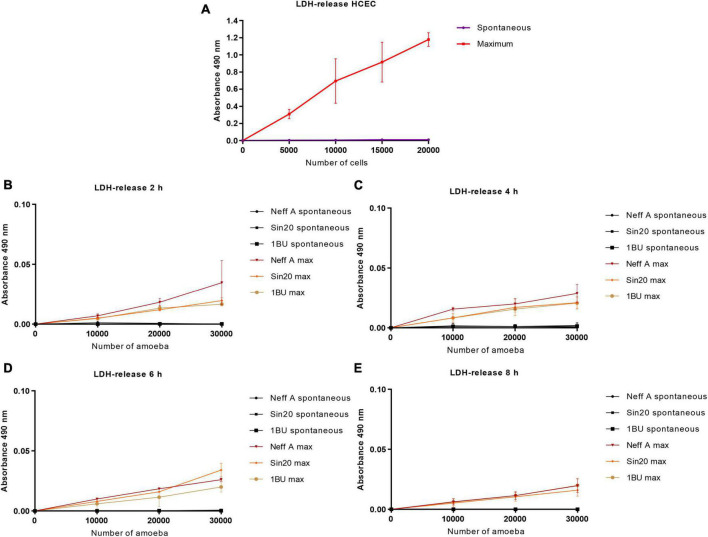
Effect of HCEC **(A)** and amoeba **(B–E)** densities on absorbance at 490 nm by using the LDH release assay, at different times. The background absorbance obtained with the media alone without cells was subtracted from all data. “Maximum” corresponds to the quantity of LDH released into the cell culture medium by lysed or damaged cells, and “Spontaneous” represents the quantity released by living or non-lysed cells.

The effect of the cell density of the *Acanthamoeba* strains Neff, SIN20, and 1BU and of the HCECs on LDH activity was assessed using a defined volume of LDH lysis buffer. As shown in Figure 2, the cell density affected the linearity of LDH activity. This effect was more pronounced with HCECs (Figure 2A) than with *Acanthamoeba* strains (Figures 2B–E). This finding may be attributed to the fact that, owing to their larger size, mammalian cells can release more LDH into the extracellular environment than amoebae. In contrast, the incubation time and type of *Acanthamoeba* strain in serum-free medium did not affect LDH release.

### 3.3. *Acanthamoeba* produced low signals in presence of tetrazolium salt and NanoLuc^®^ luciferase prosubstrate

**FIGURE 3:**
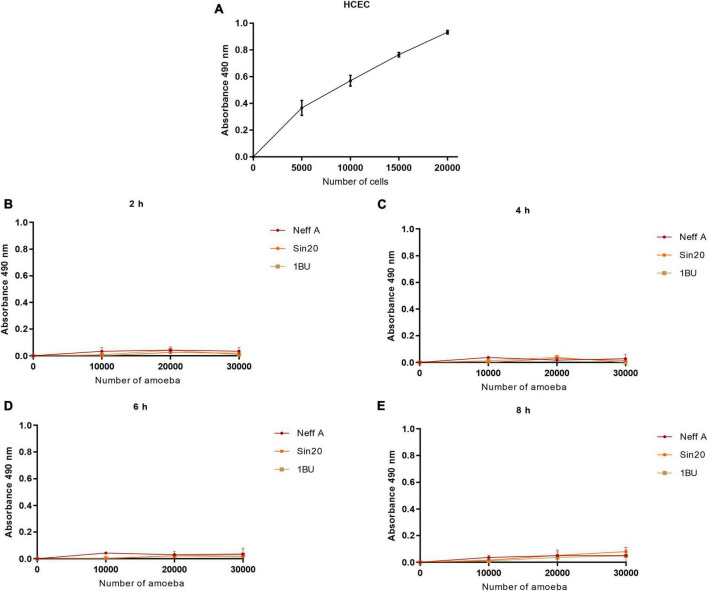
Effect of HCEC **(A)** and amoeba **(B–E)** densities on absorbance at 490 nm by using the MTS assay, at different times. The background absorbance obtained with the media alone without cells was subtracted from all data.

**FIGURE 4:**
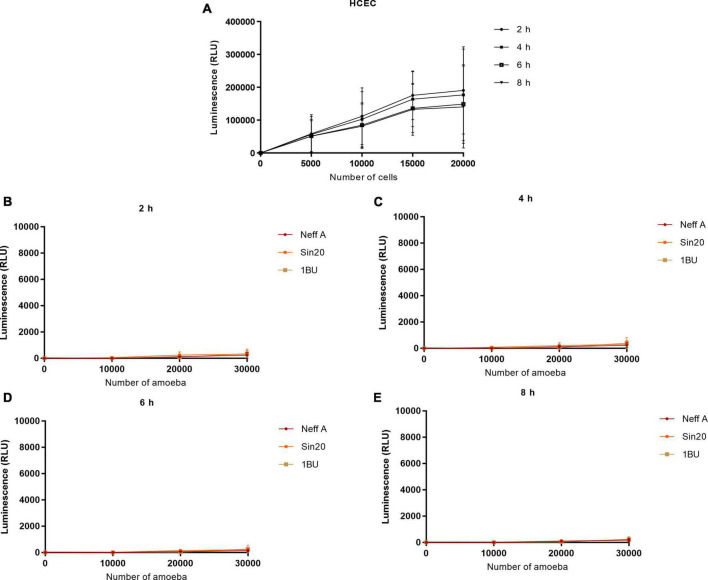
Effect of HCEC **(A)** and amoeba **(B–E)** densities on the luminescence signal by using the RealTime-Glo™ MT Cell Viability assay. The background absorbance obtained with the media alone without cells was subtracted from all data.

Similarly, the effect of cell number on absorbance at 490 nm and bioluminescence was evaluated using the MTS and RealTime-Glo™ MT Cell Viability assays, respectively, (Figures 3, 4). In contrast to the observations made in the LDH assay, *Acanthamoeba* did not increase the linear response between cell density and absorbance at 490 nm and bioluminescence, respectively, suggesting the inability of *Acanthamoeba* to considerably reduce the tetrazolium salt and NanoLuc^®^ luciferase prosubstrate, respectively, and to produce a strong signal. However, a strong signal was observed with HCECs (Figures 3A, 4A). No differences in linearity were observed between the different *Acanthamoeba* strains used in this study.

### 3.4. Cytotoxic effects of *Acanthamoeba* on human corneal epithelial cells during co-culture

Subsequently, the cytotoxic effects of *Acanthamoeba* on HCECs and the effects on their viability at various incubation periods (2, 4, 6, and 8 h) and different MOIs (MOI 1, MOI 2, and MOI 3; see Table 1) were evaluated using the cytotoxicity assays. All assays were performed according to the manufacturer’s instructions, and wells containing only serum-free medium were used as controls to account for culture medium background absorbance and luminescence.

**FIGURE 5:**
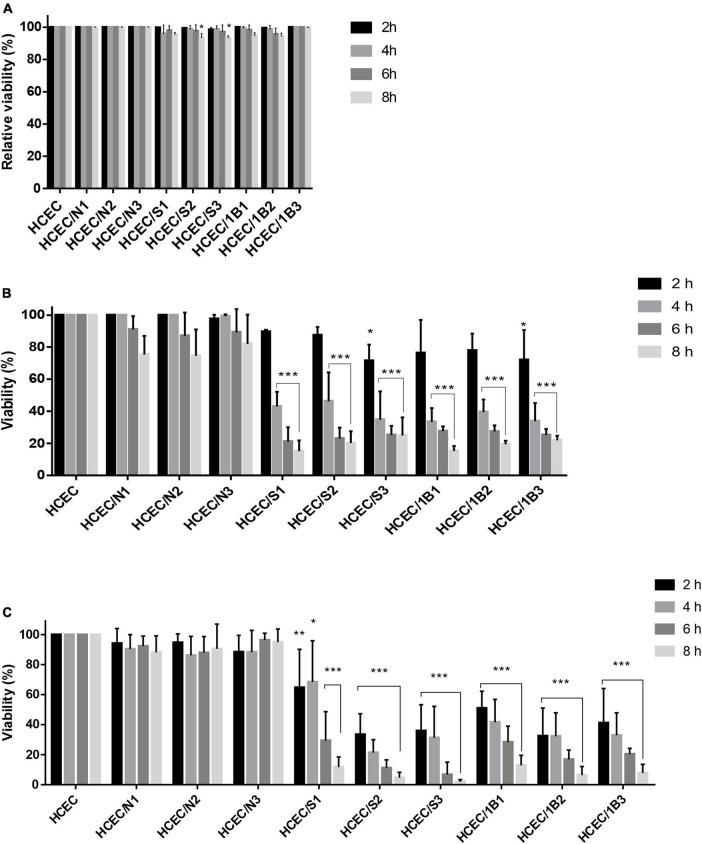
LDH release **(A)**, MTS **(B)** and NanoLuc^®^ Luciferase prosubstrate assays **(C)**. Percentage of the viability of HCEC alone and after co-culture with *Acanthamoeba* strains Neff A (N), SIN20 (S) and 1BU (B) for different time periods (2, 4, 6, and 8 h), and MOIs (MOI 1: N1, S1, and B1; MOI 2: N2, S2, and B2; MOI 3: N3, S3, and B3). Values represent the means of three independent experiments, each in triplicate. Data were plotted after the correction of the media background. Statistical analysis was performed through two-way ANOVA with Dunnett’s multiple comparisons test (*P < 0.01, **P < 0.001, and ***P < 0.0001).

As shown in Figure 5A, in the LDH assay to evaluate *Acanthamoeba*-induced cytotoxicity in HCECs, no significant differences were observed in the viability of HCECs inoculated with the environmental Neff and pathogenic 1BU strains and incubated for different periods. HCECs co-cultured with the SIN20 strain showed a slight difference in the viability of the mammalian cells at MOI 2 and MOI 3 after 8 h incubation, which refers to minor cytotoxic effects of this strain, but no significant differences were observed at other MOIs and incubation times.

The MTS and RealTime-Glo™ Cell Viability assays were used to assess the effect of *Acanthamoeba* on the viability of HCECs in co-culture. Both pathogenic strains showed a considerable effect on the viability of HCECs in both assays, except at MOI 1 and MOI 2 after 2 h incubation in the MTS assay (Figures 5B, C). The percentage of viability of HCECs in contact with pathogenic strains 1BU and SIN20 was markedly reduced after 4, 6, and 8 h of incubation in the MTS assay and at all incubation periods in the RealTime-Glo™ Cell Viability assay (except for 1BU at MOI 1 and 4 h incubation). No significant differences were observed in the viability of HCECs in contact with the non-pathogenic Neff strain in both assays.

### 3.5. Microscopic examination and consistency with cytotoxicity assays

**FIGURE 6:**
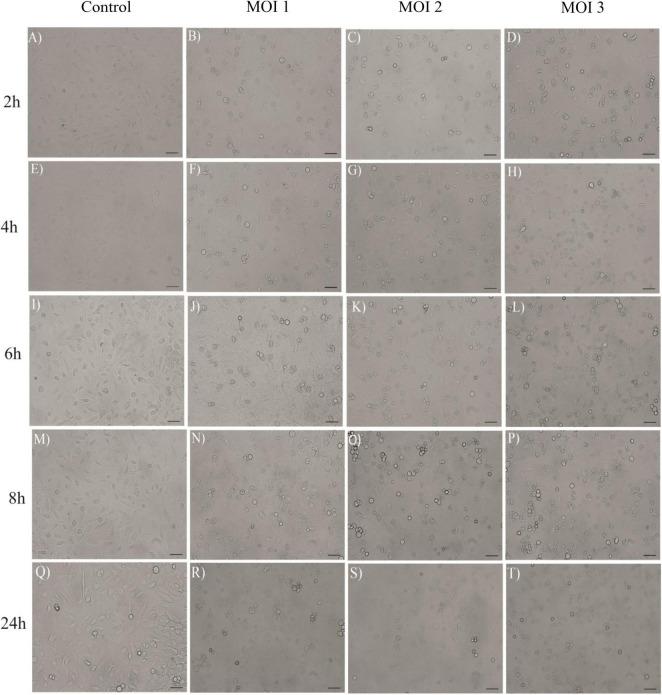
Effect of *Acanthamoeba* strain SIN20 after co-culture with HCECs, at MOI 1 **(B,F,J, N,R)**, MOI 2 **(C,G,K,O,S)** and MOI 3 **(D,H,L,P,T)** and at different times (2, 4, 6, 8, and 24 h). Panels **(A,E,I,M,Q)** represent the HCEC alone without co-culture. Scale bar = 50 μm. See Supplementary material for the amoeba controls.

The monolayer integrity of HCECs after inoculation with *Acanthamoeba* and incubation was assessed on the basis of cell detachment by phase contrast microscopy, and microphotographs were taken (Figure 6 and Supplementary material). Under all conditions, the microscopic estimation was consistent with the results obtained with the MTS and RealTime-Glo™ MT Cell Viability assays, but not with the LDH assay. The presence of cell aggregates was observed after 2 h of co-culture, and was more pronounced after 8 h of co-culture, especially at MOI 2 and MOI 3 (Figure 6). Moreover, microscopic observations after 24 h showed total or at least significant destruction of the HCEC monolayer at MOI 2 and MOI 3, while at MOI 1, numerous HCECs were still intact in co-culture (Figure 6). Owing to the difficulty in distinguishing between non-viable HCECs and non-viable *Acanthamoeba* SIN20, cell death could not be determined by cell counting using trypan blue staining.

### 3.6. Monitoring *Acanthamoeba* cytotoxicity

After inoculation and co-culture of *Acanthamoeba* with HCECs, significant differences in results were observed between conventional LDH assay and other methods used in this study (Figure 5). Results of the MTS and RealTime-Glo™ MT Cell Viability assays, which were consistent with the microscopic observations of cell detachment, showed that the viability of HCECs was substantially affected by the presence of pathogenic *Acanthamoeba* strains, whereas almost no difference was observed with the LDH assay.

## 4. Discussion

Three different cytotoxicity assays, namely the LDH, MTS and RealTime-Glo™ MT Cell Viability assays, were compared in terms of their sensitivity in detecting and measuring *Acanthamoeba* cytotoxicity on human corneal epithelial cells (HCECs), in combination with microscopic examination. It was shown that pathogenic *A. castellanii* significantly reduced HCECs viability in a dose-dependent manner starting from 2 h of co-culture at varying ratios, and this effect could be monitored reliably with the MTS and RealTime-Glo™ MT Cell Viability assays, well corresponding with the observation of cell detachment and destruction.

Various methods to monitor and evaluate *in vitro* cell viability and cytotoxicity are available, such as colorimetric and luminescence assays and microscopic examination in combination with staining and cell counting. The methods compared in the present study, except for the rather new RealTime-Glo™ MT Cell Viability method, have been extensively used during recent years to evaluate the cytotoxicity of novel antimicrobial agents against protozoan parasites such as *Leishmania*, *Toxoplasma gondii*, *Naegleria fowleri* or also *Acanthamoeba* (Ganguly et al., 2006; Jin et al., 2009; Jha et al., 2015; López-Arencibia et al., 2015; Colon et al., 2019). The LDH assay is typically used to identify host cells that have lost membrane integrity and are considered dead on the basis of LDH release into the cell culture medium; in contrast, the MTS and RealTime-Glo™ MT Cell Viability assays use compounds that are metabolized and reduced by viable mammalian cells.

In this study, we first evaluated whether the number of amoebae (0–30,000 per well) and HCECs (0–20,000 per well) influenced the signal obtained with the LDH, MTS, and RealTime-Glo™ MT Cell Viability assays (Figures 2–4). Although HCECs produced a strong signal in all assays, this was not observed with the amoebae. While the LDH assay showed a cell density-dependent signal also for the amoebae, the MTS and RealTime-Glo™ MT Cell Viability assays showed very weak signals for various concentrations of amoebae and incubation periods, indicating that the amoebae poorly metabolized the substrates. The MTS results are consistent with a previous study, in which the density of *Acanthamoeba castellanii* also did not affect the tetrazolium salt reduction and produced a weak signal (Heredero-Bermejo et al., 2013). No differences in the activity of LDH released and reduction of tetrazolium salt and NanoLuc^®^ luciferase prosubstrate were observed between the *Acanthamoeba* strains investigated.

Subsequently, the cytotoxic effects of the different *Acanthamoeba* strains on HCECs were assessed using the above-mentioned tests. Interestingly, there was a striking discrepancy between the cytotoxicity results obtained with the LDH assay and the results obtained with the two other assays as well as with the microscopic examination of cell detachment. While generally, the cytotoxicity measured with the LDH assay was similar to that reported for *A. castellanii* on HCECs in a previous study (Sohn et al., 2019), we found that the LDH assay underestimated the proportion of dead cells and overestimated the proportion of living cells and thus was not reliable to determine the cytotoxic effect of *Acanthamoeba* on HCECs. However, the LDH assay has also been used to evaluate the activity of potentially anti-amoebic agents, because this enzyme is released into the culture medium when the cell membrane integrity is affected (Sissons et al., 2005; Lorenzo-Morales et al., 2010; Anwar et al., 2018; Shi et al., 2020; Akbar et al., 2022). The results obtained in the current study corroborate the suitability of the LDH assay for evaluating *Acanthamoeba* viability, but not when *Acanthamoeba* is co-cultured with other cells. We assume that proteases produced by the pathogenic *Acanthamoeba* strains acted on the LDH released by the HCECs and thereby led to inaccurate results. A similar effect was reported for pathogenic bacteria in a recent study, where proteases produced by the bacteria during host–pathogen co-culture interacted with the LDH and thus lead to an underestimation of bacterial cytotoxicity (Van den Bossche et al., 2020). However, further studies will be necessary to confirm this hypothesis. In contrast to the LDH assay, the MTS and RealTime-Glo™ MT Cell Viability assays provided reliable and comparable results for *Acanthamoeba* cytotoxicity. The RealTime-Glo™ MT Cell Viability assay was more sensitive in determining the cytotoxic effect of *Acanthamoeba*; the lowest percentage of cell viability detected with the RealTime-Glo™ MT Cell Viability assay and MTS assay was approximately 2% with the SIN20 strain and 15% with the 1BU strain, respectively. Good correlation in significant findings in both pathogenic strains was observed between the two assays, especially after 4 h of co-culture with HCECs (*p* < 0.0001).

The ability of *Acanthamoeba* to establish contact with and adhere to host cells and induce apoptosis is crucial for their pathogenicity, but varies dramatically between strains. We used *Acanthamoeba* strains belonging to the genotype T4, since it is the most frequently genotype isolated from keratitis and non-keratitis diseases cases (Maciver et al., 2013; Walochnik et al., 2015; Castro-Artavia et al., 2017). Strains 1BU and SIN20 are AK-causing clinical isolates, isolated from patients with severe keratitis, while the Neff strain (ATCC 50373) is a non-virulent environmental isolate; and this is in line with the variations in the results obtained between 1BU and SIN20, on one hand, and Neff strains, on the other hand.

Usually, the presence of proteases and the production of extracellular vesicles are used to determine the pathogenicity of *Acanthamoeba* isolates (Khan et al., 2000; Walochnik and Duchêne, 2016; Moreira et al., 2020). The MTS and RealTime-Glo™ MT Cell Viability assays can be applied as valuable screening tools to assess directly the pathogenicity of *Acanthamoeba* isolates from environmental and clinical sources, in co-culture with mammalian cell lines.

In conclusion, the present study demonstrates the importance of choosing the most suitable method in accordance with the research purpose to meaningfully quantify cell viability and cytotoxicity *in vitro*. While the LDH assay, in contrast to the MTS and the RealTime-Glo™ MT Cell Viability assays, is suitable to determine *Acanthamoeba* viability in axenic culture, it is unsuitable to determine the cytotoxic effect of *Acanthamoeba* on host cells. However, the tetrazolium- and luciferase prosubstrate-based assays, although and because unsuitable to evaluate *Acanthamoeba* viability, were found to sensitively and reliably assess the cytotoxic effects of *Acanthamoeba* on human cells *in vitro*. To the best of our knowledge, this is the first report of a potential interference between proteases produced by *Acanthamoeba* with the LDH assay during *Acanthamoeba*–host cell co-culture leading to an underestimation of the cytotoxic effect.

## Data availability statement

The original contributions presented in this study are included in the article/Supplementary material, further inquiries can be directed to the corresponding author.

## Author contributions

JW, ALM, and TM-P designed the study. ALM, IL-M, IH-B, RM, and TM-P collected and analyzed the data. ALM, IL-M, TM-P, and JW wrote the manuscript. All authors discussed the data, read, edited, and approved the current version of the manuscript.
