# Supplementary material for: Assessing Acanthamoeba cytotoxicity: comparison of common cell viability assays
Source: Front Microbiol. 2023 Apr 25;14:1175469. doi: 10.3389/fmicb.2023.1175469 (PMC10167018; doi:10.3389/fmicb.2023.1175469)
Supplement: Supplementary file 1 [file Data_Sheet_1.docx]

Supplementary Material

Assessing Acanthamoeba-host cytotoxicity: comparison of common cell viability assays

Alvie Loufouma Mbouaka, Iwona Lesiak-Markowicz, Irene Heredero-Bermejo, Rounik Mazumdar, Julia Walochnik *, Tania Martín-Pérez

*** Correspondence:** Julia Walochnik: julia.walochnik@meduniwien.ac.at

## Supplementary Figures

A)


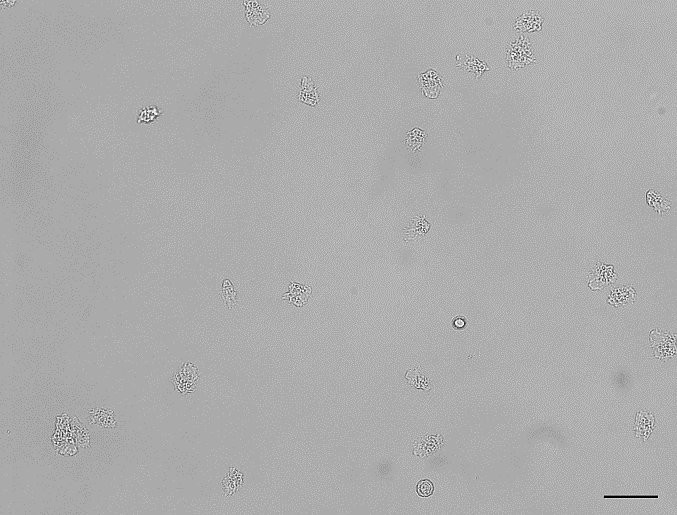


B)


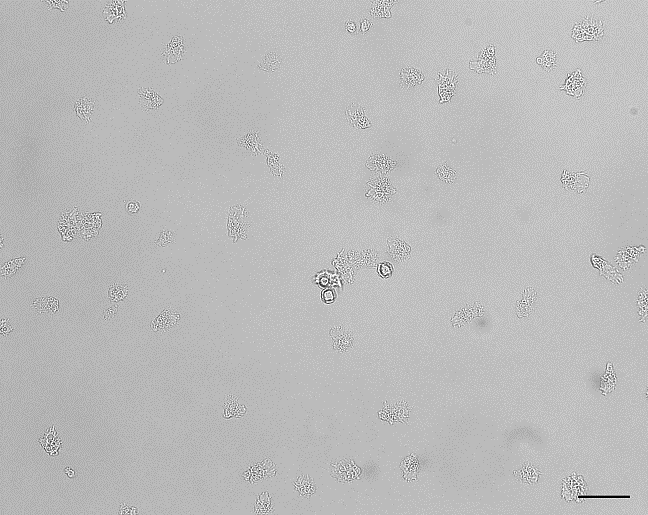


C)


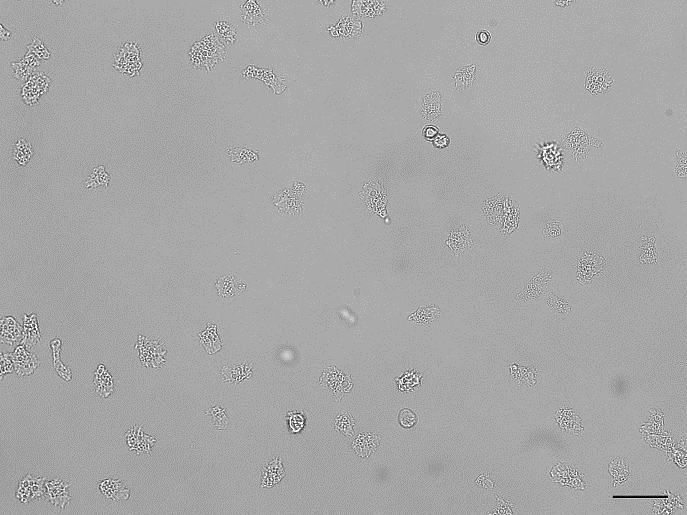


D)


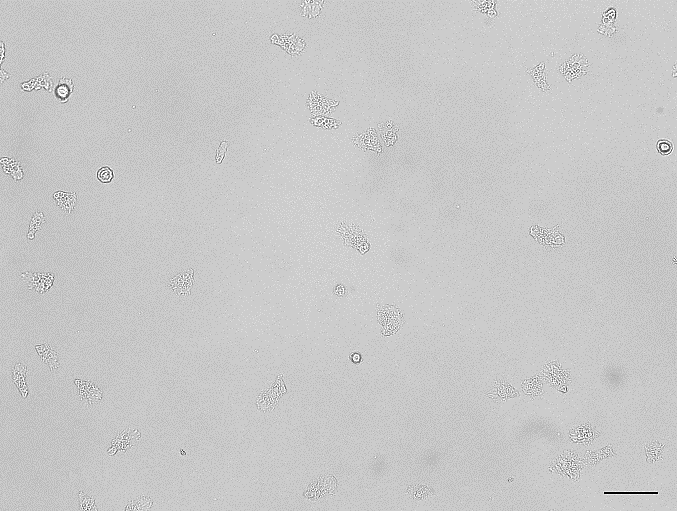


E)


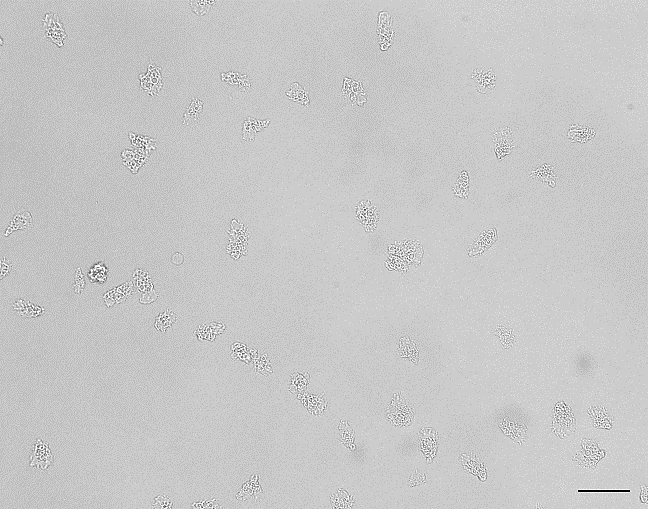


F)


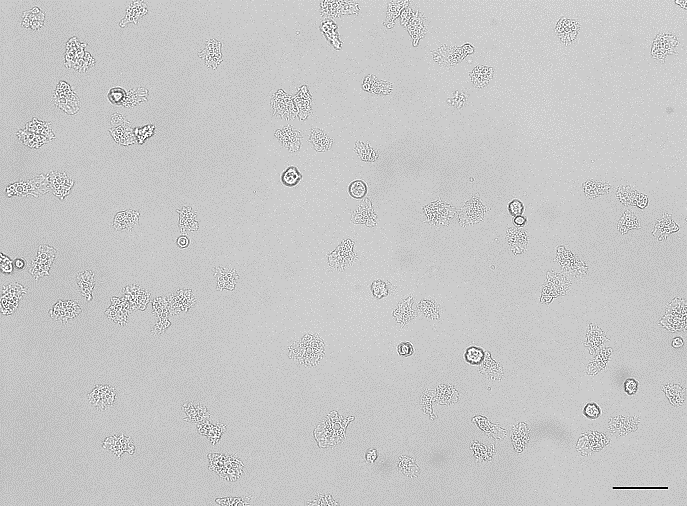


G)


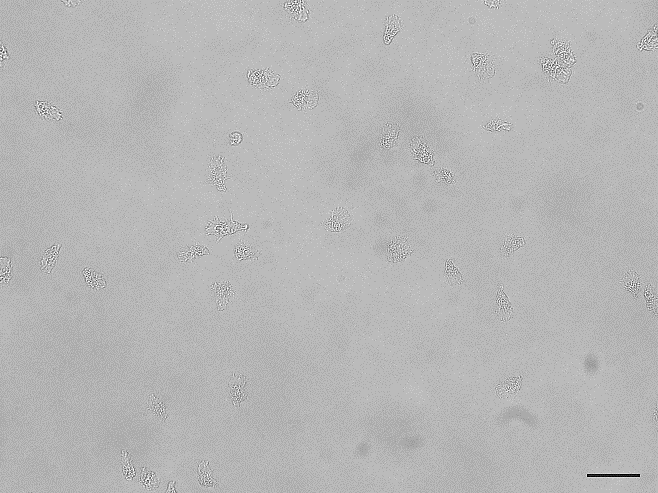


H)


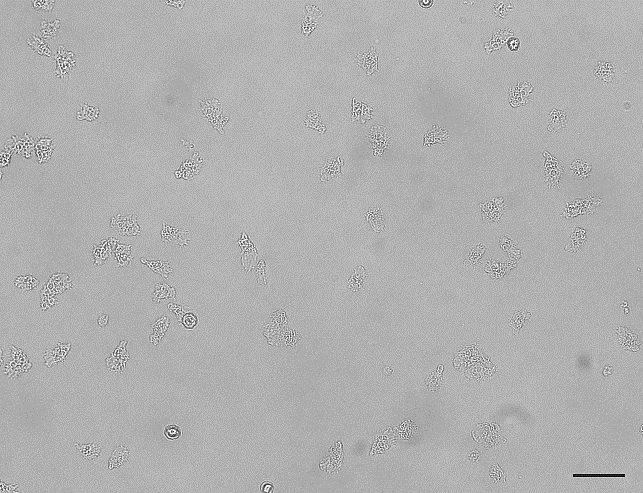


I)


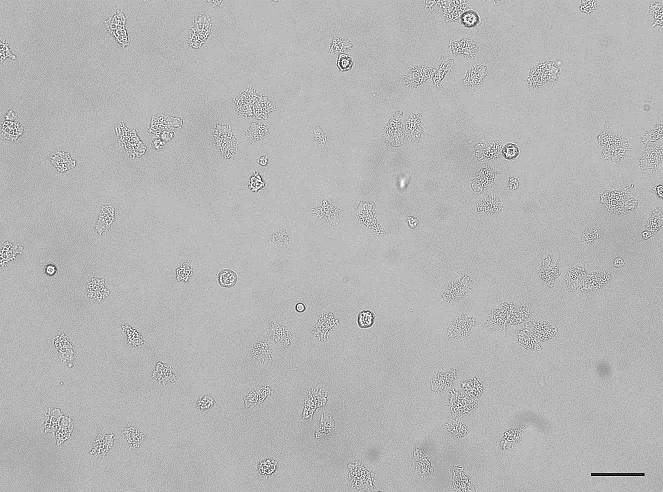


J)


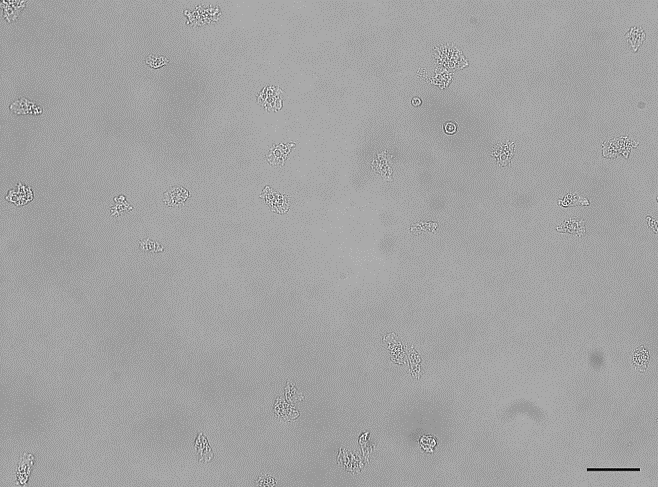


K)


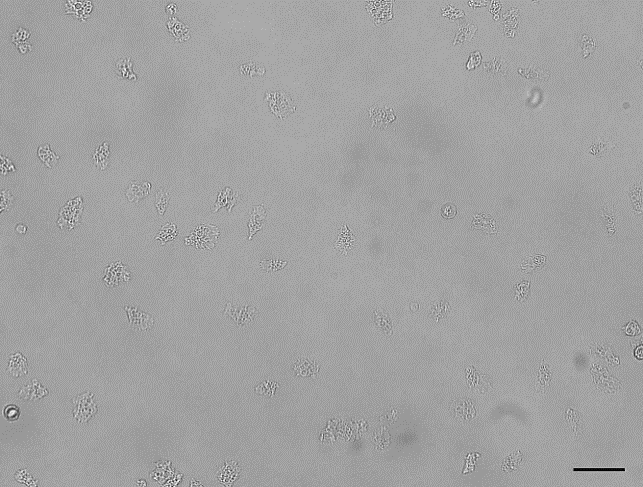


L)


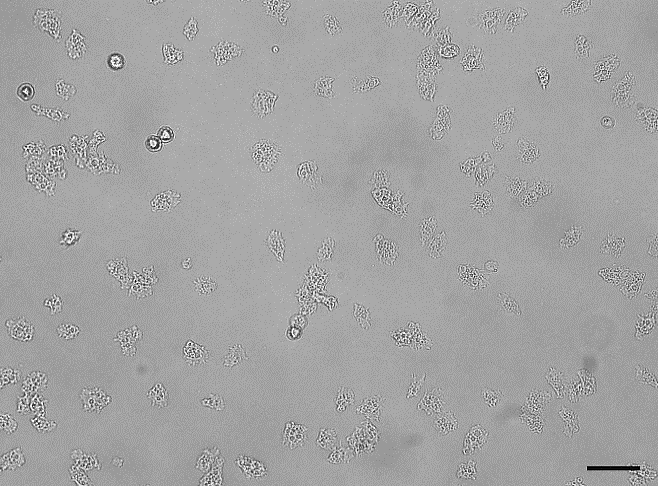


M)


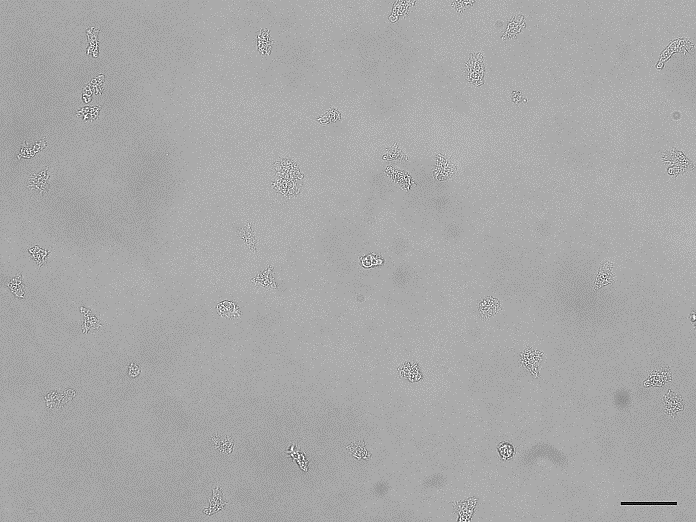


N)


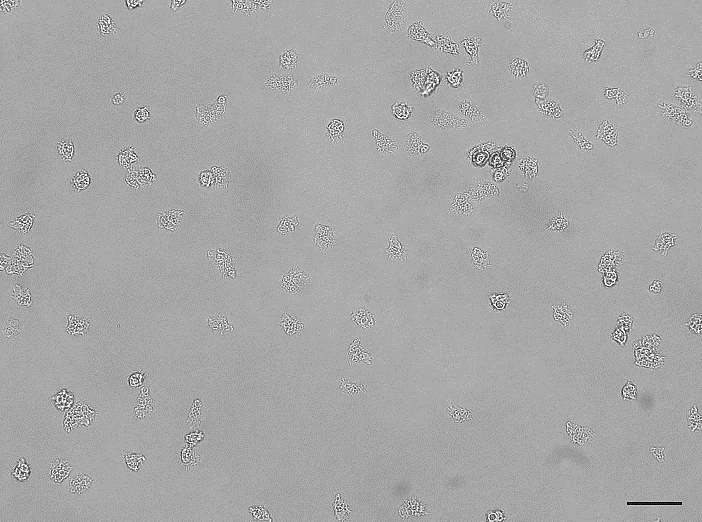


O)


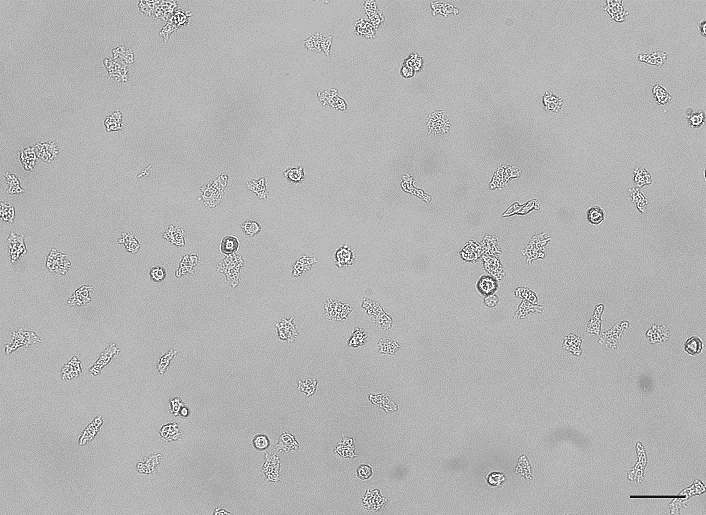


**Supplementary Figure 1.**

Microphotographs. Amoeba controls inoculate with a density of 5000 (A, D, G, J, M), 10000 (B, E, H, K, N) and 15000 ( C, F, I, L, O) cells, and incubated for 2h (A, B, C), 4h (D, E, F), 6h (G, H, I), 8h (J, K, L) and 24h (M, N, O). Scale bar = 50µm
